# An immunologic portrait of cancer

**DOI:** 10.1186/1479-5876-9-146

**Published:** 2011-08-29

**Authors:** Maria Libera Ascierto, Valeria De Giorgi, Qiuzhen Liu, Davide Bedognetti, Tara L Spivey, Daniela Murtas, Lorenzo Uccellini, Ben D Ayotte, David F Stroncek, Lotfi Chouchane, Masoud H Manjili, Ena Wang, Francesco M Marincola

**Affiliations:** 1 Infectious Disease and Immunogenetics Section (IDIS), Department of Transfusion Medicine, Clinical Center and Trans-NIH Center for Human Immunology (CHI), National Institutes of Health, Bethesda, Maryland, 20892, USA; 2 Department of Internal Medicine, University of Genoa, Italy; 3 Center of Excellence for Biomedical Research (CEBR), Genoa, Italy; 4 Department of Oncology, Biology and Genetics and National Cancer Research Institute of Genoa, Italy; 5 Department of Biology, Northern Michigan University, Marquette, MI 49855,USA; 6 Weill Cornell Medical College in Qatar, Education City, Doha Qatar Box 24144; 7 Department of Microbiology & Immunology, Virginia Commonwealth University Massey Cancer Center, Richmond, VA 23298, USA

## Abstract

The advent of high-throughput technology challenges the traditional histopathological classification of cancer, and proposes new taxonomies derived from global transcriptional patterns. Although most of these molecular re-classifications did not endure the test of time, they provided bulk of new information that can reframe our understanding of human cancer biology. Here, we focus on an immunologic interpretation of cancer that segregates oncogenic processes independent from their tissue derivation into at least two categories of which one bears the footprints of immune activation. Several observations describe a cancer phenotype where the expression of interferon stimulated genes and immune effector mechanisms reflect patterns commonly observed during the inflammatory response against pathogens, which leads to elimination of infected cells. As these signatures are observed in growing cancers, they are not sufficient to entirely clear the organism of neoplastic cells but they sustain, as in chronic infections, a self-perpetuating inflammatory process. Yet, several studies determined an association between this inflammatory status and a favorable natural history of the disease or a better responsiveness to cancer immune therapy. Moreover, these signatures overlap with those observed during immune-mediated cancer rejection and, more broadly, immune-mediated tissue-specific destruction in other immune pathologies. Thus, a discussion concerning this cancer phenotype is warranted as it remains unknown why it occurs in immune competent hosts. It also remains uncertain whether a genetically determined response of the host to its own cancer, the genetic makeup of the neoplastic process or a combination of both drives the inflammatory process. Here we reflect on commonalities and discrepancies among studies and on the genetic or somatic conditions that may cause this schism in cancer behavior.

## Introduction

It was about a decade ago when the first studies attempting to re-classify cancer according to global transcript analysis were published [1-3]. A countless number of publications followed attempting to reshape the landscape of cancer based on transcriptional or other high-throughput platforms for better diagnosis, prognosis and prediction of cancer behavior. As the galore of expectations triggered by these investigations is settling into a more realistic perspective, consistent observations are emerging from the bulk of information that sparkle novel insights on the biology of cancer. These observations describe a cancer phenotype characterized by immune effector mechanisms that are commonly observed during acute inflammation.

Under certain conditions, inflammation seems to promote carcinogenesis, whereas in other situations it seems to have anti-tumor effects. The intensity and nature of the inflammation could explain this apparent contradiction [4,5]. In most cases, the inflammation associated with cancer is similar to that seen with chronic inflammation, characterized by the production of growth and angiogenic factors that stimulate tissue repair and growth. Macrophages are the primary source of secreted pro-inflammatory cytokines and tumor macrophage infiltration has been shown to be an independent predictor of poor prognosis in malignancies such as lymphoma, non small cell lung cancer and hepatocellular carcinoma [6-8]. Occasionally, however, it is observed a cancer inflammatory process, similar to acute inflammatory processes, characterized by the presence of innate and adaptive T cell responses which favors an immune effector mechanism capable of inducing spontaneous or treatment-induced cancer regression. In this process the presence of intrinsic immune signatures reminiscent of an anti-viral state is painting a new portrait of cancer [9]. This portrait includes paradoxical relationships between the role of immunity in modulating cancer growth versus rejection [4,10-13]. Furthermore, the canonical role played by the immune system in recognizing and clearing aberrant tissues broadens its functions to modulate tissue regeneration, angiogenesis and pro- or anti-apoptotic mechanisms, which in turn may affect directly or indirectly the natural history of cancer [14-16].

### Immune signatures in melanoma

**Figure 1:**
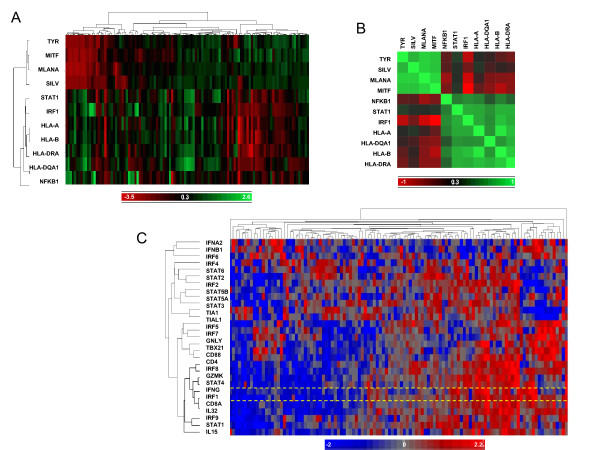
**Transcriptional profiling of 114 melanoma metastases; A) self organizing heat map displaying selected IRF-1 related transcripts together with the expression of MITF and melanoma differentiation antigens**. Each column represents a melanoma metastasis. The dendrogram indicates the degree of similarity among genes (rows) or melanoma samples (columns) using Pearson's correlation coefficient. B) Matrix similarity based on Pearson correlation for the same genes. C) Self organizing map of IRF transcripts with related genes that have been observed to be expressed in tumors displaying better prognosis [23,24,45] and expressed during immune-mediated tissue-specific destruction [9,10]. Highlighted is the IFN-γ/STAT-1/IRF-1/IL-15 cluster associated with a Th1 type of immune response. The yellow dashed lines underline the close relationship between the expression of IFN-γ and IRF-1 in the tumor microenvironment.

Long ago, it was suggested that spontaneous regression or involution of malignant melanoma could be explained in terms of cellular immunity [17]. Cochran AJ [18] observed in 1969 that about 37% of primary melanomas displayed a lymphocyte aggregation at their periphery and an additional 35% displayed a "striking mixture of lymphocytes and plasma cells"; local recurrence occurred significantly less frequently in patients showing a mixed lymphocyte/plasma cell response. Based on a large study, Clemente CG et al. [19] conclusively reported in 1996 that the presence of tumor infiltrating lymphocytes in the vertical growth phase of primary cutaneous melanoma was an independently favorable prognostic factor. The immune-active phenotype of melanoma is not limited to primary lesions. Years ago, we observed that metastases from cutaneous melanoma could be segregated into two subclasses according to the coordinate expression of transcripts annotated with innate and adaptive immune function [20,21]. The transcriptional profile of "immune active" metastases kept apart from that of normal melanocytes when compared to the transcriptional profile of immunologically "quiescent" metastases. Moreover, when serial biopsies were performed on the same metastatic lesions, a unilateral shift was noted from the quiescent to the immune active phenotype [21]. Finally, we observed that the expression of melanoma differentiation antigens was inversely correlated to the expression of immune-related transcripts supporting a de-differentiated state occurring at a later stage of disease rather than a distinct taxonomy [22]. We recently confirmed this observation by assessing the transcriptional profiles of 114 melanoma metastases (Figure 1A). As previously observed an Interferon (IFN)-y type signature with enhancement of the expression of Interferon regulatory factor (IRF-1), antigen processing and presentation genes was frequently found to be inversely correlated with the expression of the microphthalmia-associated transcription (MITF)-cluster of melanoma differentiation antigens and cancer testis antigens (Figure 1B). Moreover gene expression analysis of 17 breast tumor specimens with at least 10% infiltrating cells indicated a similar behavior compared to melanomas suggesting a portrait ascribable to negative selection of cancer cells simultaneously expressing the antigens target of immune recognition and the corresponding antigen presenting molecules. Alternatively, progressive de-differentiation could be associated with an autochthonous enhancement of constitutive metabolic functions encompassing constitutive immune activation. Activation of IRF-1 in the same melanomas correlated with that of transcripts associated with improved survival in cancer [23,24] (Figure 1C) and immune-mediated tissue specific destruction [9,10]. Based on the same cohort of patients we also analyzed whether there was an association between the expression of immune gene and signatures involved in damage associated molecular pattern (DAMP) such as High mobility group box 1 (HMGB1)[25]. The absence of any correlation between them suggest, at least in this context, that the presence of immune effector molecules in tumor side and in tumor microenvironment is not due to the presence of sterile inflammation events or activation of ischemia-related signature occurring in tumor site (data not shown).

Prospective molecular profiling of melanoma metastases undergoing immunotherapy with the systemic administration of high dose interleukin (IL)-2 suggested that lesions likely to undergo complete regression were immunologically activated before therapy [21]. Recently, in a pilot study of 19 patients vaccinated with a combination of four tumor antigens plus IL-12, Gajewski et al. [26] observed by global transcriptional analysis that tumors of patients who respond to therapy display a pre-existing inflamed status characterized by the expression of interferon stimulated genes (ISGs). Moreover, expression of T cell attracting chemokines such as CCL2/MCP-1, -3/MIP1α, -4/MIP1β, -5/RANTES and CXCL-9/Mig and -10/IP-10 was observed at the transcriptional and subsequent [27] protein level. These findings were associated with the histopathological demonstration of a brisk infiltrate of CD8+ T cells in the same tumors. The role of these chemokines in recruiting activated effector T cells was confirmed *in vitro *and in reconstituted xenografts. Interestingly, together with the effector component of the immune response, the inflamed tumor phenotype also displayed the presence of immune inhibitory mechanisms including the expression of indoleamine-2,3-dioexygenase, PD-L1 and T regulatory cells. These findings suggest that tumors of the inflamed phenotype do not discriminate among various components of the immune response as they can sustain immune effector and immune regulatory functions simultaneously; it is probably the overall balance between the two vectors that determines the ultimate fate of these cancers individual cancers. More recently, the same group identified a similar pattern predictive of favorable outcome in patients vaccinated with dendritic cell loaded with multiple tumor antigen-derived peptides [28]. Similar findings were observed by GSK-Biologics in non small cell lung cancer and melanoma patients undergoing vaccination with MAGE-3 protein [29,30]. An inflammatory phenotype was also found to be predictive of good outcome in patients with melanoma treated with IL-2 [21,31] or anti-CTLA-4 mAb [32]. Moschos et al [33] observed a higher density of dendritic cells and T cells in melanomas of patients who exhibited clinical response to adjuvant high dose IFN-α therapy; the same group identified a higher expression of phosphorylated STAT1 tyr^701 ^(pSTAT1) and higher pSTAT1/pSTAT3tyr^705 ^in cancer cells of patients with tumors displaying this type of infiltrate and this was associated with longer overall survival [34]. Treatment with IFN-α further enhanced the levels of pSTAT1 while decreasing those of pSTAT3 and total STAT3 in tumor cells further altering the balance between the two transcription factors in favor of the pro-inflammatory and pro-apoptotic phenotype [35-37]. As a functional correlate, it was observed that increased pSTAT1/pSTAT3 ratios were associated with down-stream increase in expression of ISGs by tumor cells including tapasin 2, a protein whose expression is tightly regulated down-stream of pSTAT1 signaling through the master regulator of the acute inflammatory process IRF-1 [9]. Similar variation in constitutive levels of pSTAT-1 could be observed in melanoma cells by Lesinski et al [38] who also observed that pSTAT1/pSTAT3 levels were inversely affected by IFN-α treatment; these enhanced ratios correlated with anti-proliferative effects of IFN-α.

Interestingly, others did not observe such a relationship between presence of tumor infiltrating lymphocytes and improved survival in patients with metastatic melanoma although the absence of tumor infiltrating lymphocytes predicted the development of lymph node metastases [39].

### Immune signatures in other cancers

The dichotomy between an immunologically active or a quiescent cancer phenotype is not exclusive of melanoma. Several studies described a neoplastic phenotype enriched in immunologic signatures or immune infiltrates that could be observed in cancer of ovarian [40-44], colon [23,44-53], breast [54-56], hepatocellular [44], prostatic [55-60], pancreatic [44,61], pulmonary [55,62,63], renal [64], head and neck [65,66], urotelial cells [67], anal squamous cells [68] and skin [69] origin. According to most studies, presence of immune infiltrate and/or transcriptional evidence of immune activation correlated with good prognosis (Table 1).

**Table 1:** Immune cellular and molecular patters associated with outcome in cancer

	T cell infiltrate	STAT-1 IRF-1 T-bet^+^ IFN-γ ISGs	GNLY GZM TIA	CXCL-9 CXCL-10 CXCL-11 CXCR3	CCL-5 CCR5	References*
**Melanoma**						
*Better Survival*	*+*					[18,19]
*Response to ITx*	*+*	*+*	*+*	*+*	*+*	[21,26,27,30,33]
**Ovarian Cancer**						
*Better Survival*	*+*		*+*	*+*		[40-44,70-77,83]
**Colorectal Cancer**						
***Better Survival***	*+*	*+*	*+*	*+*	*+*	[23,44-46,49-51,53,130]
**Breast Cancer**						
*Response to CTx*	*+*			*+*		[54]
*Better Survival*	*+*	*+*				[24,55,81]
**Lung/Bladder/Prostate/Cancer**						
*Better Survival*	*+*					[62,63,67]
**Renal Cancer**						
*Better Survival*	*+*			*+*		[64]
**Skin/Head and Neck/Anal Squamous Cell Carcinoma**						
*Better Survival*	*+*	*+*		*+*		[65,66,68,69]
**Esophageal Cancer**						
*Suppression of tumor growth*		*+*				[96]
*Better survival*	*+*					[131]

CTx chemotherapy; ISGs = Interferon stimulated genes; ITx = Immunotherapy

The four categories of immune functions selected for this table summarize the most commonly observed transcriptional patterns associated with the immunologic constant of rejection [9,10]

*Manuscript s proposing at least one of the categories presented in the Table as a predictor of outcome; for more detail see text.

T cells represent the dominant immune infiltrate in ovarian cancer and the presence of CD8-expressing T cells within the intra-epithelial compartment is a strong predictor of improved survival [40,43,70]. Although most ovarian cancers harbor T cells in the surrounding stroma, approximately 50% of them are infiltrated in the intra-epithelial compartment. Independent of response to chemotherapy, when this infiltrate is present, the five year survival is 35% compared to 4.5%. Several studies have confirmed these findings in various ethnic groups [71-77] providing indisputable evidence that T cell infiltration is strongly associated with an improved survival in ovarian cancer. As pointed out by Kandalaft and Coukos [70], it remains, however, unclear whether the T cell infiltrate bears a causative effect on the improved survival by effectively eliminating tumor cells or rather represents a sign of indolent tumor cell biology characterized by slower growth that enhances the chance for immune cells to infiltrate the tumor microenvironment. This hypothesis is challenged by the observation that tumors with a potentially higher proliferation index are in general those characterized by higher T cell infiltrate [74]. This may be due to a higher mutational rate of these tumors that results in higher immunogenicity due to the expression of neo-antigens [78]; this phenomenon has been also hypothesized for BRCA1 in breast and colon cancers in which microsatellite instability induces more undifferentiated tumors and denser T cell infiltrates. Alternatively, tumor-reactive T cells may have dual function inhibiting tumor growth and at the same time inducing epigenetic changes in the tumor cells that leads to tumor escape. For instance, we have shown that C producing T cells can induce methylation of the neu promoter, resulting in neu antigen loss and tumor relapse [79]. Recently, we observed an inverse correlation between HER-2/neu-specific immune responses and HER-2/neu expression in the tumors of patients with breast carcinoma. Such inverse correlation was associated with nuclear translocation of IFN-γ Ra in the tumor cells [80]. However, despite the increased tumor differentiation and malignancy, the greater presence of T cell infiltrated is associated with overall favorable prognosis [81,82].

Intra-epithelial T cell infiltrates in ovarian cancer have been associated with enhanced expression of IFN-α, IL-2 and HLA class I molecules [40,83] suggesting that the T cell infiltrate is associated with activation of a Th1 type of immune responses. More recently, it was observed that ovarian and other cancers can be heavily infiltrated with IL-17-producing T cells and their presence is often accompanied by that of IFN-α/IL-2-expressing T cells [44]. It was observed that IL17 and IFN-α synergistically stimulate the production of CXCL-9/Mig, -10/IP-10 and -11/ITAC chemokines in correlation with strong T cell infiltrate [40]. These chemokines primarily attract activated T cells expressing their receptor CXCR3 [84]. Other chemokines including CCL21/SLC/Exodus2 and CCL22/MDC were highly expressed in tumors with immune infiltrates, while an inverse correlation between presence of IL-17-expressing T cells and regulatory T cells was observed. Overall, the combined expression of IFN-γ and IL-17 expressing T cells was predictive of improved survival. As discussed later it was also observed that the presence of IL-17-expressing T cells was due to the secretion of IL-1β and IL-23 p19 by tumor infiltrating macrophages. This positive role of IL-23 p19 on survival of patients with ovarian cancer was also reported by an independent group which observed that high IL-12 p35 and IL-23 p19 transcriptional levels were associated with better outcome in ovarian cancer [85].

In 1998, Naito et al reported a correlation between infiltration of colon cancers by CD8 expressing T cells and improved survival [50]. It was subsequently recognized that CD8+, CD45RO+, CD68+ T cells were mostly present in the cases with good prognoses [51]. Recent investigations of the primary tumor microenvironment in colorectal cancer allowed the uncovering of four major intra-tumor immune profiles respectively characterized by: 1) a strong and coordinated cytotoxic Th1 immune phenotype expressing CD8+, CD45RO+ T cells, the transcription factor T-box protein 21 (T-bet), interferon regulatory factor (IRF)-1, interferon (IFN)-γ, granulysin and granzyme-B; 2) tumor angiogenesis (VEGF); 3) non-coordinated immune responses and, 4) a weak immune reactive phenotype [14,45]. The first transcriptional phenotype corresponded to immunohistochemical evidence of CD8 T cell infiltration and was characterized by good prognostic significance when the T cells were present both in the center and in the invasive margins of the tumors. These signatures were associated with lack of expression of histological markers associated with metastatic behavior such as vascular emboli, lymphatic invasion and peri-neural invasion (collectively termed VELIPI). This re-classification was more reliable in predicting disease outcome than conventional TNM staging. It was later observed that the expression of the aforementioned Th1 type genes is tightly regulated probably by the expression of IFN-γ [23]. Interestingly, recent independent studies observed a paradoxical association between the infiltration of colon cancer by T cells expressing the T regulatory cell marker FoxP3 [49,52,86,87] or PD-1 [88] and favorable prognosis in patients undergoing chemo- or chemo-immunotherapy. This observation is in line with the previously described association between a longer disease free survival and the presence of FoxP3 T regulatory cells in head and neck and ovarian cancer. As no transcriptional analysis was performed in these studies, it is unknown whether this represents a distinct cancer phenotype or rather that the expression of FoxP3 is also, though paradoxically, a component of the more broadly described immune phenotype. More recently Tosolini et al., by profiling colon cancer biopsies, described two clusters of genes associated with regulatory functions. Although the first cluster (IL-10/TGFβ) was not associated with a favorable outcome, the FoxP3 (second cluster) mRNA expression and the presence of high density FoxP3 positive cells were associated with better survival [53]. The same observation was made in a recent study whereon patients with metastatic melanoma received high-dose interleukin-2 plus the gp100:209-217(210M) peptide vaccine. The vaccine plus interleukin-2 group, as compared with the interleukin-2 only group, experienced a significant improvement in overall clinical response and longer progression-free survival. It was also noted an increase in CD4+FOXP3+ T cells in patients who responded to therapy independent of the treatment received. It was hypothesized that the increased levels of T regulatory cells in patients who had a response to treatment represent a counter regulatory response after a strong anti-tumor immune reaction [89].

In addition, the evidence that regulatory T cells can lose FoxP3 [90], effector T cells can transiently express FoXP3 without acquisition of suppressive functions [91,92] and FoxP3 acts as tumor suppressor gene and it is expressed on tumor cells [93-95] complicates the interpretation of the aforementioned studies in absence of functional and cell-specific analyses.

**Figure 2:**
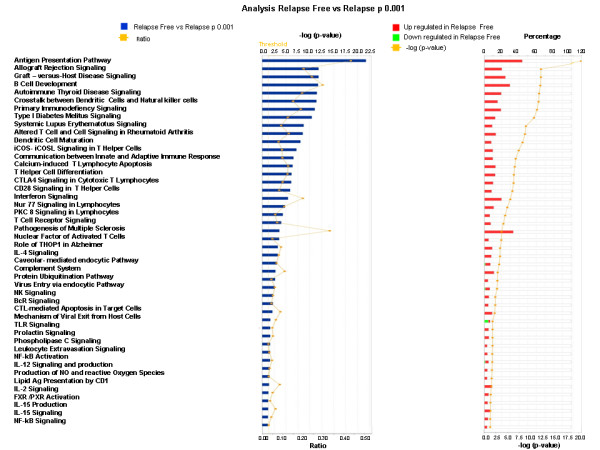
**Functional gene network analysis based on 349 genes derived from a Student's t test comparing relapse free and relapse patients affected by breast cancer **[24]. Ingenuity pathway analysis of canonical pathways over-expressed in relapse free breast cancer patients compared to patients who did not relapse identified 46 pathways significantly affected. Immune function genes involved in interferon signaling associated with allograft rejection, autoimmune reaction, antigen presentation, B cell development and NK Cell Signaling ranked at the top and were up-regulated in lesions of breast cancer patients who endured a relapse-free survival.

A similar portrait was recently described for breast cancer [54]. Moreover, transcriptional analysis of primary breast tumors bearing at least a 10% immune cell infiltrate detected a set of genes with immune function that predicted recurrence free survival. Among them, a 5-gene signature including IGKC, GBP1, STAT1, IGLL5, and OCLN predicted relapse-free survival with higher than 85% accuracy [24]. The extended signature associated with relapse-free survival included transcripts dependent upon interferon signaling which had been previously associated with antigen presentation, allograft rejection, autoimmunity, B cell development and natural killer cell signaling. Interestingly, genes involved in primary immunodeficiency signaling, T cell apoptosis, CTLA4 signaling and production of NO and reactive oxygen species were also up-regulated in the tumor specimens of relapse-free patients (Figure 2). Such paradoxical findings as to simultaneous up-regulation of immune effector genes and immune suppressor genes may suggest that tumor-derived factors were responsible for the expression of immune suppressor genes thereby facilitating cancer progression even in the presence of the increased immune effector genes. However, removal of breast tumors by conventional therapy must have eliminated the source of immune suppressive factors and resulted in down-regulation of the suppressor genes; subsequently, the immune effector genes may have protected the patients from their residual micro-metastases and relapse.

Furthermore, Denkert et al [54] observed that a robust pre-existing intra-tumor and stromal lymphocytic infiltration was a significant independent predictor of complete response to anthracycline/taxane neo-adjuvant chemotherapy. In particular, this study identified CXCL9/Mig to be strongly associated with intra-tumor lymphocytic infiltrate and better responsiveness in all cohorts of patients studied. Furthermore, statistical evaluation suggested that the response to chemotherapy was dependent on lymphocytic infiltrates and that CXCL9/Mig expression varied gradually among lesions as a continuous parameter. Conversely, elevated expression of IRF-2 (an inhibitor of IRF-1 transcriptional activity) and high IRF-2/IRF-1 ratios were observed to be associated with worse prognosis in esophageal cancer [96]. Since IRF-1 is a central modulator of the expression of the CXCR3 ligand chemokines, this study reinforces the relevance of the IFN-γ/STAT-1/IRF-1 axis as a favorable prognostic factor in cancer.

Besides the preclinical evidence suggesting that anticancer immune responses contribute to the success of chemotherapy, Ladoire S. et al [97] observed that also the nature of tumour-infiltrating lymphocytes after neoadjuvant chemotherapy could be used as a predictive factor for survival of breast cancer patients. Studying a series of 111 consecutive HER2- and a series of 51 non-HER2-overexpressing breast cancer patients treated by neoadjuvant chemotherapy, it was observed that high CD8 and low FOXP3 cell infiltrates after chemotherapy were significantly associated with improved relapse free survival (p = 0.02) and OS (p = 0.002), and this predictive pair outperformed classical predictive factors in a multivariate analysis.

Interestingly, an older study observed an opposite pattern whereby lymphocytic infiltrate was associated with worse 5 year disease-free survival and with reduced estrogen receptor expression [98]. However, in that case, no functional assessment of the immune infiltrate could be inferred through transcriptional signatures. Additionally, the down regulation of estrogen receptor genes in samples with lymphocytic infiltration could have driven the results, since the estrogen receptor negative tumors are insensitive to endocrine therapy. In fact, the hormonal treatment can dramatically improve the prognosis of the estrogen receptor positive tumors, thereby counterbalancing the negative effect of the absence of tumor infiltrating lymphocytes [99].

Wallace et al. [58] reported signatures related to activation of interferon stimulated genes (ISGs) in primary prostate cancer of patients of African American ancestry; these signatures, contrary to what observed by previous groups in other cancers, were associated with a worse prognosis in individuals of this ethnic background compared to European Americans. However, although a common theme related to enhanced interferon signaling was observed by both groups, the latter did not observe activation of the typical inflammatory genes described by the former centered on the IFN-γ/IRF-1 pathway rather signatures more typically related to signaling down-stream of type 1 IFNs such as IFN-α or -β and to the induction of angiogenesis. In fact, immunohistochemical analysis demonstrated that these signatures where associated with enhanced angiogenesis and, contrary to the colon study, infiltration of tumor associated macrophages rather than T cells; these observations are, therefore, opposite of those observed in colon cancer suggesting that these immune active prostate cancers have a VELIPI phenotype. Moreover, the same group observed a immune phenotype of basal-like breast cancer portraying a higher frequency of FoxP3-expressing T regulatory cells and characterized by poorer prognosis [56]. It was also observed that upon separation of tumor epithelium from tumor stroma by micro-dissection, the immune signatures were predominantly expressed by the latter. These observations suggest that the cancer immune phenotype comes in different flavors which can be partly attributed to a mixed activation of pathways associated to type II or type I IFN signaling and possibly the latter displays a less clear prognostic value.

### On the origin of immune signatures

Together with gene expression profiling, immunohistochemical (IHC) validation of gene expression data and tissue microarrays (TMAs) are useful tools to clarify the prognostic relevance of gene expression in discrete tumor tissues [100,101]. However, IHC and TMAs techniques are limited by the subjectivity of scoring methods compared to transcriptional profile which give objective or quantitative information that can be normalized to standard reference samples.

Although gene expression analysis has proven accurate in the characterization of the tissue microenvironments supporting the relevance of ongoing immune responses, it cannot provide information about the source cell expressing immune effectors gene. Based on transcriptional evidence, it is generally assumed that signatures of immune activation identified by testing whole cancer tissue reflect activation of genes expressed by infiltrating immune cells. However, we recently compared immunologic signatures of primary pancreatic cancer tissues pancreatic cancer cell lines and observed activation of innate immunity including activation of ISGs in both [61]. Moreover, primary xenografts displayed a similar dichotomy between an immune active and an inactive phenotype. Immunohistochemistry analysis of primary tumor lesions confirmed the expression of MxA, a typical ISGs. Thus, it is likely that the immune signatures expressed by cancer tissues are, at least in part, due to the activation of immune mechanisms intrinsic to the tumor cell biology. Indeed, others have observed signs of immune activation in cultured melanoma cell lines which displayed constitutive pSTATs activation [38]. Similarly, Zeimet et al [102] in a study including 138 ovarian cancer samples observed high levels of IRF-1 expression in cancer cells; the high expression of IRF-1 by cancer cells was associated with enhanced presence of CD3+ T cell which only occasionally stained positively for IRF-1. In this case, however, it is difficult to predict whether IRF-1 expression was secondary to the secretion of IFN-γ by the T cells, which, in turn, are insensitive to autocrine activation due to down-regulation of the IFN-γ receptor [103]. Thus, the best evidence that the immune signatures observed in the tumor microenvironment are, at least in part, driven by the intrinsic cancer cell biology resides in the constitutive activation of immune genes observed in resting cancer cells *in vitro*. It is interesting to note that the constitutive activation of cancer cells observed *in vitro *through the detection of the phosphorylation of STAT-1 is generally modest; yet it corresponds to a transcriptional profile fully associated with the coordinate expression of ISGs activated downstream of the STAT-1/IRF-1 axis. Moreover, it appears that this constitutive activation of pSTAT-1, though mild, predisposes cell lines to higher sensitivity to further stimulation with IFN-γ [38,104].

Currently, no correlative study has been reported to test whether the signatures observed in immune activated tumors are also present in cell lines derived from the same tumors. It is important to note, however, that expression of various transcription factors associated with IFN signaling by cancer cells has been broadly described in various tumors [35] and it has been associated with better overall survival at least in melanoma [34]. Moreover, it is believed that activation of such transcription factors such as members of the STAT family is at least in part mediated by activation of various oncoproteins involved in tyrosine kinase signaling [35,37]. Thus, although it is not clear why TIL are present in some and not all tumors, it is becoming increasingly clear that some intrinsic characteristics of the tumor cells themselves may drive in part the presence of immune infiltrates. For instance, secretion of endothelial factors and chemokines by tumor cells has been deemed responsible for this phenotype in various cancer models [27,105-107]. It is also possible that the borderline constitutive immune activation or anti-viral state of cancer cells *in vivo *perpetuate a positive feedback loop whereby the cancer cells not only invite immune cells to the tumor microenvironment but they are also more sensitive to pre-inflammatory factors secreted by immune cells. A recent commentary outlines different characteristics of the tumor cells that may explain paradoxical observations on tumor infiltrating cells of the immune system [108].

### Prognostic significance of immune signatures

Constitutive production of immune stimulatory molecules and activation of immune mechanisms in cancer cells is likely to drive the homing of immune cell within the tumor microenvironment as recently demonstrated in experimental models [27,109]. It is also possible that the constitutive immune activation of cancer cells makes them more sensitive to immune stimulatory mechanisms induced by TIL as suggested by the enhanced expression of pSTAT-1 in response to IFN-α stimulation [38]. However, the activation of innate immune mechanisms may have broader implications than those predicted by direct immune interactions. IRF-1 and IRF-5 for instance are powerful pro-apoptotic transcriptional activators which directly inhibit the cyto-protective activity of other pro-survival factors such as NF-*k*B [110-112]. Moreover, IRF-1 inhibits VEGF expression thereby exhibits anti-angiogenic effects [113]. Thus, the paradoxical observation that signatures of actively growing tumors are similar to signatures of immune-mediated tissue-specific destruction during acute rejection [9,10] may be explained by a borderline situation in which immune activation is not sufficient to activate all the mechanisms required to clear the organism of cancer cells; however, these tumors are more susceptible to fluctuations of immune functions driven by environmental conditions such as sporadic viral infections or the pro-inflammatory effects of chemotherapy [114] or immune therapy [21] that can suddenly shift the balance toward partial or complete tumor elimination.

Constitutive expression and activation of STAT-3, a common oncogenic signaling pathway, has been clearly associated with cancer progression and poor prognosis [115]. Moreover, Wang et al. [115] showed an inverse correlation between STAT-3 activation in tumor cells and expression of pro-inflammatory cytokines associated with adaptive and innate immune responses. Thus, it is possible that activation of the IFN-γ/STAT-1/IRF-1 pathways serves to counteract the constitutive activation of STAT-3 in some cancer cell lines in the context of metastatic melanoma [34]. While experimental models support this hypothesis, very little is known about the association of various STAT proteins in cancer cells with immune infiltrates.

### The mechanisms leading to immune activation of cancer cells and/or microenvironment

It could be argued that infiltration of the immune cells into the tumor site is a characteristic dictated by the genetic background of the host [116]. Indeed polymorphisms in the expression of cytokine receptors, CXCR3 and CCR5 in particular, have been implicated in the ability of the host to mount effective immune responses in several pathological conditions including allograft rejection [117]. Considering the central role that the ligands for these receptors play in immune-mediated tumor rejection, it is reasonable to speculate that genetic polymorphism may be in part responsible for the tumor infiltration of the immune cells and polymorphism of several genes could represent a multi factorial component of this phenomenon. This is just an example of the genetic variants that could affect the infiltration of tumors in some but not all patients. At the transcriptional level, expression of CXCR3 and CCR5 ligands is tightly controlled by the activation of the IFN-γ/STAT-1/IRF-1 axis; however, when the expression of the CXCL-9, CXCL-11 and CCL-5 is assessed in melanoma metastases, almost a perfect correlation is observed between them and their respective receptors CXCR3 and CCR5 expressed by tumor-infiltrating T cells and NK cells. It is possible that polymorphisms in the expression of these receptors may variably influence trafficking of the immune cells to tumors that express comparable levels of the relevant chemokine (Figure 2). Alternatively, it is possible that the presence of T cells may be dictated by factors other than the chemokines such as the expression of antigen and antigen presenting molecules that could induce the expansion and persistence of T cells reaching the tumor.

It is also possible that the genetics of the tumor may drive infiltration of different cells of the immune system to the tumor site [73,78,81,82]. For instance, it was observed that ovarian cancers with p53 mutations are more frequently infiltrated with intraepithelial T cells [73]. Although these findings could be interpreted as a higher likelihood for such tumors to express neo-antigens that could expand the tumor antigen-recognizing T cells, poorly differentiated tumors may secrete soluble factors with paracrine activity, favoring tumor growth [20].

Several studies emphasized on IRF-1 as a transcriptional activator that facilitates a Th1 immune phenotype and leads to tumor infiltration of CD8+ lymphocytes associated with a favorable prognosis in cancer patients [23,102,118]. Although the mechanisms leading to IRF-1 activation at the transcriptional level are not known, it is interesting to note that at least in ovarian cancer a convergence is observed between the immune phenotype and the expression of IFN-γ[119], IL-12 p35 and IL-23 p19 [85] which are powerful stimulators of IRF-1 expression. Moreover, Kryczek et al [44] observed a combined infiltration of IL-17 expressing CD4+ T cells and CD8+ effector T cells; through synergistic action between IL-17 and IFN-γ expression, Th17 cells were observed to stimulate the expression of CXCL-9 and -10 by ovarian cancer cells as well as tumor infiltrating macrophages in order to recruit more effector T and NK cells to the tumor microenvironment. This combination was observed to positively predict patient outcome in the context of ovarian, colon, hepatocellular and pancreatic carcinoma as well as in melanoma. Altogether this study demonstrated that Th17 cell infiltration in several tumor types was quantitatively and positively correlated with NK cell-mediated innate and adaptive immune responses. The authors investigated the role of various cell subtypes potentially responsible for the induction of this tumor phenotype and identified tumor-associated macrophages as the primary driver through the production of IL-1β, IL23 p19 but not IL-6 and transforming growth factor-β; this effect was, however, neutralized by the presence of T regulatory cells in the same environment [44].

The importance of a Th1 immune environment has also been observed by Dieu-Nosjean *et al*. [62] who observed an association between the presence of tertiary lymphoid organs and better survival outcome in patients with non small cell lung cancer; these tertiary lymph nodes were characterized by the infiltration of B cells, CD8+ and CD4+ T cells expressing T-bet and polarized toward a Th1 phenotype. This is a remarkable observation considering that tertiary lymph nodes are structurally and functionally identical to secondary lymphoid tissues and contain fully activated dendritic cells. It remains unclear why some tumors induce the formation of these tertiary lymphatic structures which are rare in normal conditions and are transiently present only in areas of massive inflammation.

What are the mechanisms leading to constitutive activation of these transcriptional factors in cancer? While strides have been made in the understanding of the activation of the JAK/STAT pathway in leukemia [120] very little is known about solid cancers. Yet, several candidate mutations have been shown to lead to the activation of innate immune mechanisms in several cancer types including melanoma. Most studies focused on constitutive activation of the master regulator or innate immunity and the cell survival factor NF-*k*B [121-123]. Since NF-kB and the IRFs are tightly involved in direct and indirect interactions [112,124-126], it is reasonable to postulate that the same mechanisms may be at the basis of the constitutive activation of STATs and in particular STAT-1 in melanoma. Yet, this information is lacking at the present time and future work should address this question.

### Correlation between immune signatures of the tumor microenvironment and systemic immune response

It has been clearly shown that patients with early stage (stage II) or more advanced cancers suffer impaired immune function. The most striking example is decreased activation of ISGs and decreased ability to phosphorylate STAT-1 by circulating immune cells [127-129]. These findings have been reproduced in various cancer types including melanoma, breast and colon cancer. It is important to note that although statistically patients with cancer have strongly reduced immune function compared to normal healthy donors there is great individual variation with overlap of response between patients and normal donors. It is possible that the immune environment of tumors may directly or indirectly influence this systemic phenomenon. It is, however, unknown whether a correlation exists between the immune phenotype of tumors and the responsiveness of peripheral immune cells to immune stimulation. Mortarini et al [129] observed that impaired STAT activation of T cells did not correlate with frequency of CD4+/CD25+/FoxP3+ T cells at the tumor site, though they did not further compare the global transcriptional patterns of the tumors. Interestingly, they also observed that sera obtained from patients with advanced melanoma inhibited IL-2-dependent STAT activation of normal donor's T cells, and a neutralizing monoclonal antibody to TGF-β1 counteracted such inhibition. Since it is well known that tumors may express this soluble factor it would be interesting to compare the status of activation of various tumors with the behavior of circulating cells to test whether the immune phenotype of tumors drives the systemic alteration of immune function associated with the cancer bearing status. Identification of a correlation would allow indirect immune phenotyping of tumors and consequently predict prognosis by testing circulating cells.

## Summary

It is becoming clear that tumors can be segregated into at least two categories independently of their histology. Of them one bears a signature consistent with a Th1 type of immune activation which in turn is associated with, lymphocytic infiltrate, better prognosis and enhanced likelihood to respond to therapy. It remains unknown, why this dichotomy occurs, whether it depends on the genetic makeup of individuals bearing the disease or it is due to somatic mutations within cancer cells. It is also unknown, whether these signatures of strong clinical relevance are reflected by changes in the peripheral circulation. We believe that future studies comparing tumor tissue characteristics with the peripheral circulation, along with clinical data will be key to better classify cancers.

## Competing interests

The authors declare that they have no competing interests.

## Authors' contributions

FMM conceived of the manuscript, and participated in its design, coordination, analysis and interpretation of literature data.

MLA participated in the acquisition, analysis and interpretation of literature data,

All the authors made intellectual contributions and have been involved in drafting the manuscript, and approved the final manuscript.
